# Utilizing NPWT improving skin graft taking in reconstruction for extended breast skin defects following mastectomy

**DOI:** 10.1002/ccr3.4716

**Published:** 2021-10-04

**Authors:** Chia‐Yu Kuo, Jung‐Yu Kan, Chieh‐Ni Kao, Fu Ou‐Yang, Cheng‐Che Wu, Jun‐ping Shiau, Chung‐Liang Li, Ming‐Feng Hou, Shu‐Hung Huang

**Affiliations:** ^1^ Division of Breast Surgery, Department of Surgery Kaohsiung Medical University Hospital Kaohsiung Taiwan; ^2^ Department of Surgery School of Medicine, College of Medicine Kaohsiung Medical University Kaohsiung Taiwan; ^3^ Division of Plastic Surgery, Department of Surgery Kaohsiung Medical University Hospital Kaohsiung Taiwan; ^4^ Regeneration Medicine and Cell Therapy Research Center Kaohsiung Medical University Kaohsiung Taiwan

**Keywords:** breast cancer, breast reconstruction, latissimus dorsi muscle flap, negative pressure wound therapy, split‐thickness skin grafts

## Abstract

NPWT fulfill graft taking in complex breast wounds.

## Graphical abstract

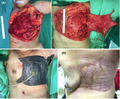

## 1 INTRODUCTION

Split‐thickness skin grafts and latissimus dorsi muscle flap are common methods for reconstructing wound defects after mastectomy. However, poor skin graft taking remains a challenge due to chest movements. The purpose of this study is to investigate the outcomes of an alternative method for large defects reconstruction after mastectomy by applying negative pressure wound therapy onto the grafts.

The chart recorded nine patients with advanced breast cancer, who received reconstruction with split‐thickness skin grafts and latissimus dorsi muscle flap with negative pressure wound therapy after modified radical mastectomy. After the wounds were covered with the latissimus dorsi muscle flap and split‐thickness skin grafts, negative pressure wound therapy was applied and kept at −50 mmHg in continuous mode for four days. Postoperative care included limiting the range of motion of the affected side for the first postoperative week, followed by removal of any surgical drains after drainage had decreased. They were reviewed regarding details of the defect size, graft take rate, and outcomes.

All patients had their wounds fully recovered at three‐month follow‐up. Two of them experienced seroma in the flap donor site, which was resolved by aspiration. The average of skin graft take rate was 96% at two weeks after the surgery. No major complications were observed, including major flap loss, large skin graft necrosis, or surgical site infections. Only one had partial skin graft necrosis less than 2cm^2^ at two‐week follow‐up, which recovered with wound care.

We proposed utilizing negative pressure wound therapy effectively fulfill skin graft taking in reconstruction of extended breast skin defects and should be considered for the management of these challenging wounds.

Breast cancer is the most common malignancy in women worldwide and is the second most common cause of cancer mortality in Asia.^1^ With the increasing incidence of breast cancer, surgical planning for advanced breast cancer has become a challenge because it involves the burden of resection. Breast reconstruction after mastectomy can be performed using many methods, and it depends on soft tissue defect and reconstruction timing.^2^ Autologous vascularized tissue graft for breast cancer reconstruction has been performed since the 1970s. The introduction of the latissimus dorsi (LD) flap with its overlying skin island has allowed the restoration of skin and volume loss after mastectomy. The LD flap can be harvested as muscular, musculocutaneous, or bony structures. The advantage of the LD flap is its accessibility, while its disadvantages include visible scars, contour deformity, and inadequate volume.^3^ The LD flap can have a maximum dimension of 20 cm ×25 cm, and primary donor site closure can be achieved when the width of the skin paddle is <8–9 cm.^4^, ^5^ In some cases, the defects are too large to be covered using a single flap; in such cases, skin grafts may be suitable for muscle flap coverage.

For the wound reconstruction of large skin defects, surgery with transverse rectus abdominis muscle (TRAM) flaps has demonstrated optimal results.^4^ If breast reconstruction with TRAM flaps is unsuitable for the patient, the use of an LD musculocutaneous flap with skin graft is an alternative. However, fixing the graft is difficult due to chest wall movement while breathing. To overcome this challenge, negative pressure wound therapy (NPWT) can be applied, which reduces the recipient site's hematoma formation and holds the skin graft tightly to reduce shear force. Thus, this modality can enhance the survival of skin grafts.^6^, ^7^

To the best of our knowledge, few reports have detailed the use of NPWT after breast reconstruction using LD muscle flaps and split‐thickness skin grafts (STSG). In this study, we report our experience with using NPWT after breast reconstruction with LD muscle flaps and STSG.

## 2 PATIENTS AND METHODS

This retrospective study reviewed nine patients with advanced breast cancer from June 2015 to October 2019 who received reconstruction with an LD muscle flap and STSGs, followed by NPWT. All patients underwent modified radical mastectomy (MRM) and developed skin defects of dimensions greater than 15 cm ×15 cm; eight of the nine patients received immediate reconstruction. Moreover, of the nine patients, one had undergone previous abdominal surgery, and the others refused to receive TRAM reconstruction. NPWT was applied to all patients, and the detailed procedure is described as follows.

### 2.1 Operation technique

Preoperatively, anatomical landmarks were marked, including bilateral inframammary folds, the tip of the scapula, the iliac crest, the posterior midline, and the border of the LD muscle flap. After the breast surgeon performed the MRM, we estimated the defect size and designed the LD muscle flap.

The patient was turned to the ipsilateral side of the breast, and a transverse incision was made along the bra line. After the pedicle of the thoracodorsal vessels was identified, the subcutaneous layer was dissected. The LD muscle flap was then raised through the tunnel below the axilla to cover the exposed ribs and adjacent soft tissue. The donor site was closed layer by layer after placing the drainage tubes. The skin graft harvested from the scalp or thigh area was secured to cover the LD muscle flap by using circumferential staples or through suturing to the wound bed. Usually, we use LD musculocutaneous flap for reconstruction. In this case series, the skin defects were too large to be covered. Thus, we chose LD muscle flap with STSGs for reconstruction.

Subsequently, NPWT was applied, which involved the use of sterile open‐cell foam sealed with a plastic adhesive drape and the application of controlled subatmospheric pressure to the wound. The continuous application of negative pressure was achieved using seal‐check systems. If an air leak was identified, the leakage site was repaired with a strip of the adherent dressing.

### 2.2 Postoperative settings

NPWT was applied at −50 mmHg in the continuous mode for four days, after which the wound was opened under aseptic conditions. The patient's range of movement was limited to shoulder abduction of up to 90° in the first week after treatment. The drainage tube for the LD muscle flap was removed if daily drainage amounted to less than 10 ml.

We obtained the following data by reviewing patient charts containing information on patient demographics, size of the skin graft, donor site, estimated graft acceptance based on physicians’ progress notes, length of hospital stay, presence of complications, and cosmetic results at two weeks, one month, and three months of follow‐up.

### 2.3 Statistical analysis

Computed descriptive statistics included mean standard deviation for continuous variables and percentages for categorical variables. All analyses were performed using Microsoft Excel for Windows (version 2001). Results are expressed as mean standard deviation.

## 3 RESULTS

The profiles of all nine female patients are presented in Table 1. The mean age of the patients was 64.78 years (range, 44–85 years), and their mean BMI was 27.12 kg/m^2^ (range, 21.90–35.87 kg/m^2^); one of them was obese (BMI >30). None of the patients smoked. Of the three patients with comorbidities, one had type II diabetes mellitus (DM), and the other two had received neoadjuvant chemotherapy. All of them were in the advanced stage of breast cancer and underwent unilateral MRM. Table 2 revealed the patients’ outcomes. One of them received delayed reconstruction. The mean size of the defect area after MRM and skin graft size for reconstruction were 399 and 240 cm^2^, respectively. Most of the skin graft was harvested from the scalp, followed by the thigh area. We applied NPWT at −50 mmHg in the continuous mode for four days. After two weeks, the mean graft take was 96.11%. Two of them developed seroma and required aspiration at the donor site. Partial skin graft necrosis occurred in one patient and healed after wound care.

**TABLE 1:** Patient profiles

Patient demographics, n = 9	
Age (year)	
Mean (SD)	64.78 (12.98)
Range	44–85
Sex, *n* (%)	
Male	0 (0)
Female	9 (100)
BMI (kg/m^2^)	
Mean (SD)	27.12 (4.24)
Range	21.90–35.87
Comorbidities, *n* (%)	
Type II diabetes mellitus	1 (11.11)
Obesity	1 (11.11)
Preoperative chemotherapy or radiation therapy	2 (22.22)
Operation methods, *n* (%)	
Modified radical mastectomy (MRM)	9 (100)
Tumor characteristics	
Stage	
3B, *n* (%)	1 (11.11)
4, *n* (%)	8 (88.89)
Recurrence	3 (33.33)
Location	
UOQ, *n* (%)	3 (33.33)
UIQ, *n* (%)	0 (0)
LOQ, *n* (%)	0 (0)
LIQ, *n* (%)	2 (22.22)
Central, *n* (%)	4 (44.44)

Abbreviations: UOQ, upper‐outer quadrat; UIQ, upper‐inner quadrat; LOQ, lower‐outer quadrat; LIQ, lower‐inner quadrat.

**TABLE 2:** Patient outcomes

Outcome	
Defect size, mean (cm^2^)	382.67
Skin graft size, mean (cm^2^)	240.00
Reconstruction	
Immediate, *n* (%)	8 (88.89)
Delay, *n* (%)	1 (11.11)
Source of STSG	
Scalp, *n* (%)	4 (44.44)
Thigh, *n* (%)	5 (55.56)
Graft take after two weeks, mean (%)	96.11
Complication	
Seroma, *n* (%)	2 (22.22)
Graft/flap partial necrosis^a^, *n* (%)	1 (11.11)

^a^ The area of graft necrosis over recipient site less than 2 cm^2^.

### 3.1 Case 1

**FIGURE 1:**
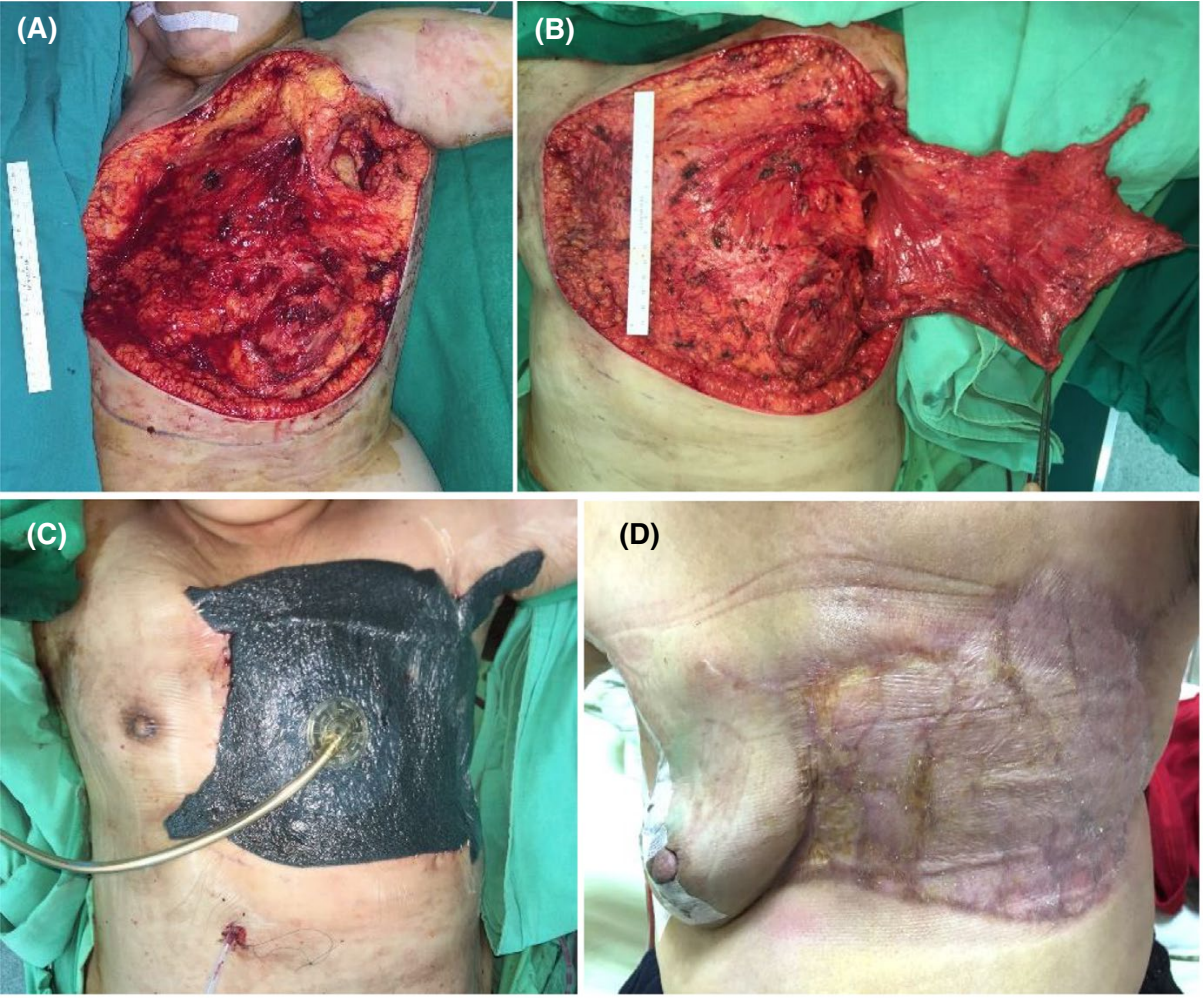
Case 1

The first case was a 59‐year‐old woman diagnosed with infiltrating ductal carcinoma of left breast, cT4bN1M1, stage IV. She had received neoadjuvant chemotherapy with epirubicin and cyclophosphamide followed by docetaxel. Surgical intervention with left MRM +axillary lymph node dissection (ALND) combined with LD muscle flap +STSG harvested from the scalp was performed smoothly (Figure 1A,1B). After immediate breast reconstruction, we applied NPWT over the entire wound at −50 mmHg in the continuous mode for four days (Figure 1C). The patient exhibited satisfactory results at 3 months after the surgery (Figure 1D).

### 3.2 Case 2

**FIGURE 2:**
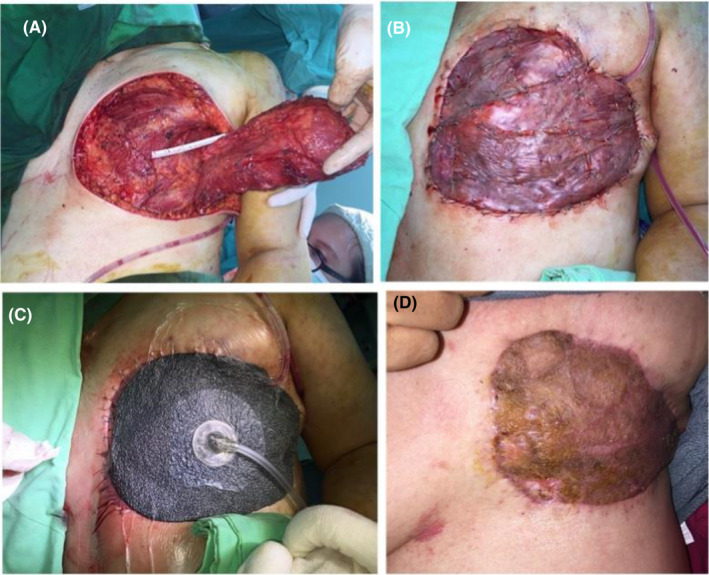
Case 2

The second patient was a 57‐year‐old woman diagnosed with invasive lobular carcinoma of left breast, cT4bN2M1, stage IV, with liver and bone metastasis. She had received neoadjuvant chemotherapy with Taxotere +Herceptin prior to the surgery. Left breast MRM +ALND was performed, combined with LD muscle flap +STSG (Figure 2A, 2B). NPWT was applied postoperatively for four days (Figure 2C). The progression was fair without any complication. The patient exhibited satisfactory results at three months after the surgery (Figure 2D).

## 4 DISCUSSION

Of the nine patients analyzed, only one had partial necrosis of skin due to poor wound healing after previous MRM with an advanced flap. All patients were treated with NPWT applied to the complex breast wound comprising LD muscle flaps and STSG skin graft. Two of the patients developed seroma over donor site, for which they received aspiration and achieved resolution at three weeks of follow‐up. Despite the presence of diabetes and morbid obesity, which were believed to be risk factors for flap failure, the patient's recovery was satisfactory.^8^

For reconstruction of large wounds in advanced breast cancer, the musculocutaneous flap provided a reliable skin island. In 1982, Hartrampf et. al. first used cranially pedicled rectus abdominis muscle flap with a horizontally oriented adipocutaneous skin island. In this technique, blood is supplied to the skin island via perforating vessels piercing the rectus abdominis muscle, arising from the deep epigastric system.^9^ Patients with advanced breast cancer are appropriate candidates for TRAM flap reconstruction.^10^ However, several complications must be considered, such as abdominal weakness, which may cause abdominal bulges or hernias.^11^ Contraindications for TRAM flap reconstruction are those who received previous abdominal surgery that may have interrupted blood supply to the flap, in addition to those unwilling to quit smoking, obese patients, and those with an uncontrollable underlying condition, such as diabetes mellitus or ongoing steroid treatment. By contrast, LD flap reconstruction presents advantages in reliability, size, and location.^12^ The dominant blood supply for the LD muscle mainly comes from thoracodorsal vessels and from perforating branches of the posterior intercostal vessels. The feasible rotation of the LD flap allows coverage of the ipsilateral side of the chest wall and the adjacent area. Because of the insufficiency of skin coverage for large defects in our patients, STSG was harvested from either the scalp or thigh for reconstruction.

Complications of LD flap reconstruction are few and may include visible scarring at the back, seroma at the donor site, shoulder functional disability, and numbness and weakness postoperatively.^13^ In our study, two patients presented with donor‐site seroma. Aspiration was performed in these patients, and the seroma gradually subsided in the following days. Partial skin island nonhealing was observed in one patient, which was resolved satisfactory through topical wound care.

As for wound healing after breast cancer surgery, prolonged wound processing may postpone further chemotherapy or radiation therapy for the patient. The acceleration of wound healing has several modalities, and the NPWT system can achieve faster wound healing, especially in difficult recipient wound beds.^14^, ^15^ Vacuum‐assisted closure is a well‐established form of NPWT, which was introduced in 1996.^16^, ^17^ The NPWT system generates negative pressure when applied to the wound through foam dressing to serve as a sealant. It promotes granulation tissue formation by increasing the blood flow, eliminates infectious debris and exudates, and facilitates the skin graft by immobilizing the graft.^14^, ^18^ Previously, NPWT was used to promote wound healing by immobilizing the graft, removing edematous fluid, increasing the blood flow, stimulating granulation tissue and neovascularization, and reducing bacterial contamination.^19^, ^20^ Nakamura et. al. conducted a retrospective study and suggested that NPWT may be superior to the tie‐over method for the stabilization of skin grafts, especially in large or muscle‐exposing defects in the trunk or extremities.^7^ NPWT yielded higher graft survival rates and shorter operative times.^7^, ^21^ We used NPWT as a graft stabilization modality to limit shear stress over the graft. The wound was opened on postoperative day four to evaluate the flap condition. All patients in our study exhibited favorable skin acceptance of the flap.

This study has several limitations. First, the reviewed literature regarding the use of NPWT in large breast wounds in combination with muscle and skin graft reconstruction is limited. Second, the sample size was small. Finally, because local recurrence is a major concern in breast cancer surgery, long‐term follow‐up is warranted in our patients.

## 5 CONCLUSION

We suggest the application of NPWT to large breast wounds after LD flap and skin graft reconstruction may be a reliable method. This study suggests it to be particularly effective in the treatment of complex breast wounds.

## CONFLICTS OF INTEREST

All authors have completed the ICMJE uniform disclosure form. The authors have no conflicts of interest to declare.

## AUTHOR CONTRIBUTIONS

Chia‐Yu Kuo, Shu‐Hung Huang, and Jung‐Yu Kan involved in conception and design. Shu‐Hung Huang, Fu Ou‐Yang, and Ming‐Feng Hou: involved in administrative support. Chia‐Yu Kuo, Shu‐Hung Huang, and Jung‐Yu Kan: involved in provision of study materials or patients. Chia‐Yu Kuo, Chieh‐Ni Kao, Cheng‐Che Wu, Jun‐ping Shiau, and Chung‐Liang Li: involved in collection and assembly of data. Chia‐Yu Kuo, Chieh‐Ni Kao, Cheng‐Che Wu, Jun‐ping Shiau, and Chung‐Liang Li: analyzed and interpreted the data. Chia‐Yu Kuo, Jung‐Yu Kan, Chieh‐Ni Kao, Fu Ou‐Yang, Cheng‐Che Wu, Jun‐ping Shiau, Chung‐Liang Li, Ming‐Feng Hou, and Shu‐Hung Huang: wrote the manuscript. Chia‐Yu Kuo, Jung‐Yu Kan, Chieh‐Ni Kao, Fu Ou‐Yang, Cheng‐Che Wu, Jun‐ping Shiau, Chung‐Liang Li, Ming‐Feng Hou, and Shu‐Hung Huang: approved the final manuscript.

## ETHICAL STATEMENT

The authors are accountable for all aspects of the work in ensuring that questions related to the accuracy or integrity of any part of the work are appropriately investigated and resolved.

## Data Availability

The author do not wish to share the patient's data. The privacy of the participant should be protected.
